# Synergy between adaptations and resilience of livelihood from climate change vulnerability: A group-wise comparison of adapters and non-adapters

**DOI:** 10.1371/journal.pone.0236794

**Published:** 2020-08-13

**Authors:** Syed Asif Ali Naqvi, Rai Hasis ul Hassan, Wenya Wu, Ashfaq Ahmad Shah, Muhammad Sohail Amjad Makhdum, Syed Ale Raza Shah

**Affiliations:** 1 Department of Economics, Government College University Faisalabad, Faisalabad, Pakistan; 2 College of Humanities and Development Studies (COHD), China Agricultural University (CAU), Beijing, China; 3 School of Management Science and Engineering, Nanjing University of Information Science and Technology (NUIST), Nanjing, China; Universitat Autonoma de Barcelona, SPAIN

## Abstract

The similarities, differences, and contradictions regarding climate change adaptation and resilience by academics and practitioners have already been documented. It is the need of time to set new precedence by observing the adaptations and resilience as tools to respond to the climate variations. This study analyzed the influence of climate change adaptations and synergy between resilience from livelihood vulnerability and adaptations. A field survey of 489 farming households is conducted with the help of a well-structured questionnaire from four districts of the south part of Punjab province of Pakistan. This study uses the Endogenous Switching Regression model for the sake of analysis. The outcomes of the study reveal that age, education, family size, total land, and seed price have significant linkage with the adoption of adaptations. The synergistic effects of adaptation and resilience are also visible here as the adaptations factors are significantly contributing towards yield, per capita income, poverty, and poverty gap of the respondents. This study suggests the provision of proper education and smart technology to help in enhancing the adaptive capacity of farmers. More imperatively, adaptations to climate variations can be concluded as a remedial tool for resilient livelihood. It is believed that the present study can be considered as a guide for future research on other regions of Pakistan and neighboring countries.

## Introduction

Climate change (CC) is one of the swiftly spread phenomena across the globe since last century and livelihood of residents of the planet is at risk [1]. One-third of the world population is directly or indirectly facing the heat of these variations [2]. The vulnerability of CC is exerting pressure on the livelihood of poor farming communities who are already on the brinks of poverty [3, 4]. The impacts fall disproportionately on resource-poor small producers who are more reliant on farming for their livelihood [5, 6].

The livelihood of rural communities is primarily based on crop production [7]. Where climate indicators play an imperative role at each stage from sowing to harvesting [8]. These variations are contributing to a reduction in the yield of almost all crops [9]. Such, impacts of CC on the productivity of crops have diversity across the regions [10]. CC is the multination issue and no country is immune to it [11]. However, countries with high income are less vulnerable to CC, in contrast, developing countries are highly vulnerable due to their inadequate capacity of technology acceptance. Although climate change is a global problem the need for adaptation is higher among developing countries where vulnerability is presumably higher as the agricultural sector is the basic source of livelihood for marginal poor rural communities [12]. A study by [13] revealed a positive impact of CC on agriculture productivity on the European agriculture sector, in contrast to it, South Asian economies like India and Pakistan are more vulnerable to climate viabilities [14].

Reduction in the yield of crops is the ample source of devastating livelihood, which causes farm income losses and poses a threat to an income threshold of daily necessities. The primary driver behind these variations is the abrupt increase in carbon dioxide (CO_2_) emission [15]. However, human activities such as industrial production, transportation, and energy consumption emit CO_2_, N_2_O and CH_4_ in the atmosphere and disturb the required complex combination of atmospheric gases balance. Therefore, industrial activities are contributing more to carbon emission [16]. However, CO_2_ is one of the most injurious greenhouse gas (GHG) for the environment [17]. GHGs backed by human activities are the key element for environmental concentration [18] and among these gases, CO_2_ contributes 63% of gaseous irradiative [19]. Developing economies are more responsible for environmental degradation (ED) as economic activities are at their boom here in these days [20], and ED, in the long run, is changing the climate pattern of the area. Urbanization is another responsible variable for ED and industrial activities and leaves serious threats to concerned communities [21].

The agriculture sector and CC have a bilateral relationship; agriculture outcomes depend upon the climate indicators, and farming practices also act as the sources of climate variability [22]. Out of the total, around 30% of GHGs are emitted by agricultural activities and at the same time, 80% of the agriculture sector is vulnerable to CC [23]. Therefore, exploring the farm income losses due to climate variations, and farmers’ efforts to averse these reductions are meaningful. Numerous kind of literature has focused on this important topic to know its widespread effects. A range of scholarly studies has explored several dimensions of CC, adaptations, and resilience on the farming sector such as [8, 15, 24, 25]. Climate-smart agriculture enhances farm yield and agricultural revenues on a sustainable basis, increase water and nutrients use efficiency, improves resilience to climatic stresses, and helps in lowering the emissions of GHG [26, 27]. Therefore, it is need of time to respond to these CCs through the latest tools like smartphone technology and updated information.

A study by [28] stated that CC has a long-term bearing on the farming sector of Pakistan and concluded that farmers are experiencing income losses and an adverse increase in the poverty levels. According to the findings of [24], CC may have a bad impact on farm outcomes and negative effects on food crops, and these results are validated by another study by [29]. A study by [30] quantified the influence of CC on the livelihood of rural households and their findings revealed that farmers, who are primarily dependent on the fruit production, are facing a decline in the farm outcomes due to climate variations and noted that extreme weather occurrences are varying the intensity in Pakistan for current and future climate scenarios. The study by [31] concluded that in general, CC is damaging the productivity of the crop of marginal growers. [32] evaluated the impact of CC on rice production and concluded that rice is more vulnerable to CC than the wheat crop concerning current and future farm production. [33] evaluated the effects of temperature and precipitation on wheat productivity and found the significant association among variation in temperature, precipitation, the area under cultivation, water, CO_2_ and crop production. [34] estimated the impact of CC on wheat production of mountainous areas of Pakistan and found the negative influence of temperature on the productivity of the wheat crop.

CC is not only impacting the farm productivities but also exerting the pressure on the livelihood of rural populations [35]. Farmers’ income is sensitive, as loss of income opens up a path for poverty and food insecurity [36]. Natural disasters also have negative impacts on rural households’ welfare [4, 37]. It can be concluded from the given literature that CC has negative impacts on farmers’ welfare. In lieu of the above discussion, it feels necessary to understand the importance of the welling of farmers’ livelihood. There is a need for the advancement in the literature to validate the performance of CC adaptations being practiced like the application of smartphone for better farming, resilience factors, and synergy existing between them. Evaluating the synergy between the said phenomena will help to understand the feedback mechanism of these strategies.

Adoption of adaptations against CC has an imperative role in compensating the farming returns [13, 23, 38, 39]. Mitigation could not be meaningful in the case of developing economies, as these cannot go toward the reduction of carbon emission due to gradual expansion in the industrial sector and urbanization. Advancement in the technology in agriculture is often assumed as an adaptation in the era of CC [40]. This study takes farmers’ responses toward predicted future CC risks and adjusting to predominant climate vulnerabilities through good practices (education, smartphone etc.) to have a resilient livelihood, as an adaptation [41]. Two types of adaptations techniques are being practiced globally, off-farm, and on-farm [42], and this study considers these both types. It is assumed that the choice of opting adaptations is based on regional and agro-ecological characteristics [43]. An adaptation measure is meaningful if it is cost-friendly for the resilient livelihood beyond climate variability [44]. According to an estimate, 300 dollars per household could be in surplus if a farmer adopts the adaptations [45]. Better crop revenues due to adaptations help in improving the material wellbeing and make farmers more resilient, and ultimately make them eligible to mitigate the risk associated with climate variations [46]. Diverse package of adaptation measures helps in attaining resilience to offset CC losses [47].

Resilience can be a prompt recovery [48] and in the present context, it is supporting farmers to recover from CC vulnerabilities. Here, in this study, we have taken farmers as the main stakeholders, who are vulnerable in the form of their yield and income losses, and worsening poverty situations. In the current scenario, farmers cannot get resilience without taking the remedial measures [49] and CC adaptations can help farmers to bounce back to their normal position.

**Fig 1:**
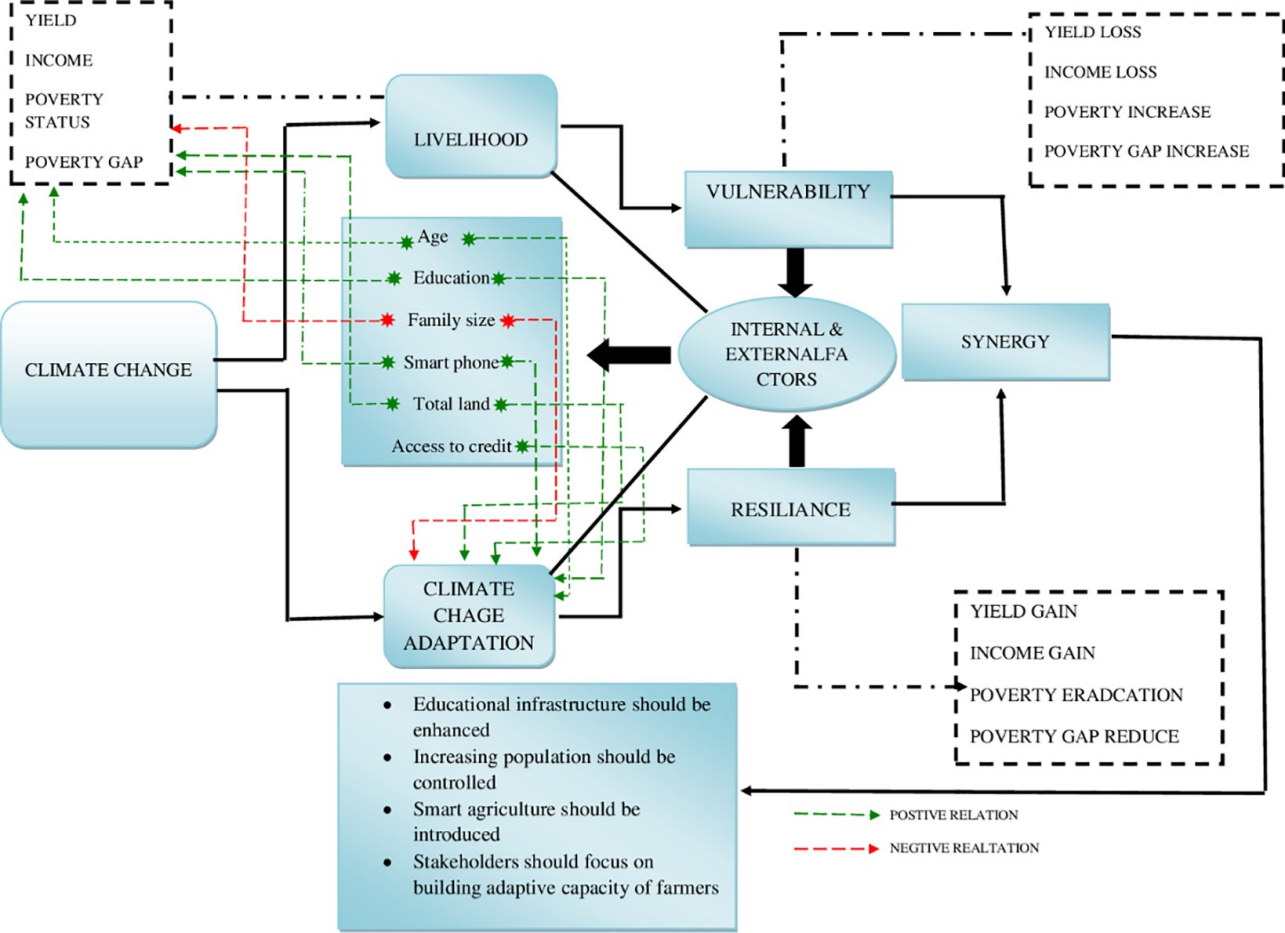
Synergy between climate change adaptations and resilience (graphical abstract).

A handsome amount of literature presents relevant contributions and most of these studies are problem and region-specific, and a research gap still exists. Several studies have focused just on the CC vulnerabilities like [3] or adaptation impacts like [11] those are conventional approaches. Although there is a long and multidisciplinary history of scientific research associated with adaptation and the definition of adaptation has varied by fields and practice [50]. This paper extends the existing literature by developing synergy between adaptation and resilience to respond properly to the variations in the context of agricultural vulnerability to climate. This study sets a new precedence by observing adaptations as a tool to combat livelihood vulnerability and developing synergy between CC adaptations and resilience through comparison of adapters and non-adapters groups. Estimation of association between adaptation and resilience will help to plan for and respond to, current and future climatic variability (see Fig 1 for details). Table 1 describes the brief review of available literature by discussing time-period, data type, the universe, methods used, and the main results of studies.

**Table 1:** Literature review.

Sr. No.	Authors	Time-period	Data type	Universe of study	Methods used	Main results
1	He et al. (2020) [1]	2019	Farm household survey	Chongqing, China	Risk-aversion experiment design	Risk cognition and adaptation cognition have significantly positive influences on CC adaptive behavior.
2	Liu et al. (2020) [7]	2018–2027	Crop cultivation and irrigation scheduling data	Northwest China	Agro-hydrological model	There would be a reduction in crop yield during the period of 2018–2027 for seed corn.
3	Mulyo and Widada (2020) [11]	2019	Farm household survey	Yogyakarta	Livelihoods vulnerability index	Level of adaptation strategies for coastal farm households is slightly better than mountainous farm households
4	Shakhawat, et al. (2020) [6]	2017	Household-level data collected	Bangladesh	Ricardian model	Current land values of farmers are sensitive to climate.
5	Jamshidi et al. (2019) [14]	1986 to 2016	Survey data, socio-demographic data	Hamadan province, Iran	Household vulnerability index	Majority of smallholder farmers are relatively vulnerable to CC.
6	Dubey and Sharma (2018) [51]	1981–2010	Simulation data	Banas River Basin	Root Mean Square Error	Crop yield of all selected crops will increase under the CC conditions in future.
7	Alam et al. (2017) [52]	2012	Households data	Malaysia	Likert scale, Analysis of variance (ANOVA)	The vulnerability of household food accessibility has increased due to CC.
8	Elum et al. (2017) [53]	2015	Household survey data	South Africa	Garrett ranking technique	Adaptation and mitigation measures help in reducing the losses from CC.
9	Mase et al. (2017) [54]	2012	Farm survey	USA	Ordinary least squares regression	Risk perceptions have a critical role in adaptation attitudes.
11	Abid et al. (2015) [55]	2014	Survey data	Punjab, Pakistan	The bottom-up approach, descriptive statistics	Limited water availability and a weak role of local government make farmers more sensitive to climate-related risks.
11	Mallari (2016) [56]	2015	Focus group discussions	Mabalacat City	Index Method	Vulnerability index map.
12	Rahman et al. (2016) [57]	2015–16	Farm survey	Costa Rican	ANOVA	Study signals the need to address the climate variations and adaptation capacity of farmers.
13	Arouri et al. (2015) [4]	2004–2010	Vietnam household living standard surveys	Vietnam	Fixed-effects regression	Households with higher mean and equal expenditure distribution are more resilient to natural disasters.
14	Ashraf et al. (2014) [58]	2011	Farm households’ survey	Baluchistan, Pakistan	Multivariate Probit model	Landholding, annual income, and farmer-to-farmer extension increase the probability of farmers’ decision to cope with hazard.
15	Bui et al. (2014) [59]	2008	Household Living standard survey	Vietnam	Fixed effects	Natural disasters worsen expenditure on poverty and inequality.
16	Moore and Lobell (2014) [60]	2030–2049	Simulation data	Europe	Biophysical modeling	There is high adaptation potential for maize to future warming.
17	Davies et al. (2013) [61]	2012–13	Phone-based or face-to-face interviews	Asia	Desk-based analysis	There is a need to tackle underlying vulnerability and the identification of several innovative multi-disciplinary approaches.
18	Joerin et al. (2012) [62]	2011	Household survey	Chennai, India	Descriptive analysis	People living near rivers and canals are at higher risk from the impacts of floods.
19	Shahid (2010) [63]	1958–2006	Meteorological data	Bangladesh	Descriptive analysis	Monsoon rainfalls have increased in the western part of Bangladesh.
20	Gbetibouo et al. (2010) [64]	1999–2008	Secondary data	South Africa	Vulnerability index	Regions most exposed to climate variability do not always overlap with those experiencing low adaptive capacity.

This study has tried to quantify the synergy existing between CC adaptations and resilience from the livelihood vulnerability. As it is clear from the above table that previous studies mostly discussed the CC adaptation based on households’ perceptions or secondary data, and to date, no study has investigated farmers’ adaptive behavior and allied synergy with CC resilience that could influence their choices. Further, this article has also attempted to answer the following research important questions, is there any significant variance between the farm incomes of adapters and non-adapters? Either, socio-economic indicators of adapters are getting better than the non-adapters, or not? Considering the cited research questions above, this study has two-fold objectives. In the first, the influence of CC adaptations on the wellbeing of rural households is quantified and secondly, we estimated the synergy between adaptations and resilience of rural households from climate variability. Outcomes from this study could contribute to an understanding of how to better coordinate the relative strengths and contributions of adaptations with the resilient behaviours to cope on-ground challenges of climate variations, as measures of increased resilience enhance adaptive capacity and decrease the vulnerability of farmers. After the essential part of the introduction and review of the literature, the remaining paper is arranged as follows: Section 2 elucidates the methodology and data used in the study. Section 3 and 4 are about assessed results and their discussion, and finally, section 5 concludes the problem under discussion based on outcomes and provides policy lessons for the solution of the issue.

## Materials and methods

### Data

To ascertain the objectives of the study, the farm-level household survey is conducted in the rural areas of Punjab province. We selected Punjab as the study universe as it has significant importance for Pakistan’s economy, particularly, its agricultural share in national Gross Domestic Product. Therefore, four districts namely Bhakkar, Khanewal, Multan, and Muzzafargarrh from the southern part of Punjab province of Pakistan are taken in the sample. The selected sampling units have diverse climate conditions and cropping patterns. Household heads (HHH) or representatives of farm households are interviewed for the data collection. As the population is heterogeneous, this is why multi-stage stratified random sampling technique is employed for the data gathering. In the first stage, four districts are selected and then in the second stage, two Tehsils from each district are taken. In the next stage, four villages from each tehsil are selected. Sixteen growers are randomly selected from each village. Cross-sectional data employed for the study were collected in June–July 2019 with the help of a team of enumerators who were trained before the survey. A pilot survey was conducted to remove the discrepancies and loopholes to produce a better quality of data. In actual, 512 farmers were selected for the interviews and out of this sample, 489 farmers were considered as valid respondents.

As this study is about the economic implications, it is why Ethical clearance is not taken. In Pakistan, ethical clearance body (National Bioethics Committee) issues clearance number to basic sciences experiments oftenly, containing human or animal tissues etc. However, authors and enumerators have taken verbal consent from the study participants before the conductance of the survey. Participants were briefed that their data would only be used for the study purpose and they agreed to give the required information. The authors declared that they did not have any conflict of interests.

However, we faced rejection from a few farmers but those were replaced. A brief and well-structured questionnaire was developed and farmers were asked about; socio-economic and farm characteristics, CC awareness, and vulnerability according to their perspective, institutional facilities, and CC adaptations. Information on farm households, agricultural practices, production and costs, access to extension, social networking, overtime climate-related changes, and allied risks, adaptations to climate change, access to credit, farm and household assets, other income sources, and other were collected during the survey. The study employed both qualitative and quantitative data collection for a deep understanding of the topic. Datasets show that farmers are practicing more than one adaptation measures. Study has taken two types of the farmers, one group is using the adaptation measures to cope with the climate-related issues and consider them as adapters, and another group of respondents is not using these techniques (non-adapters). However, the use of a mixture of on and off-farm adaptations is also reported. In the study area, the main adaptation techniques being practiced include crop diversification, use of the hybrid seed, and cultivation of supplementary crops to support the input cost of the main crop. The questionnaire used for the study purpose has been provided in the supporting information (S 1 Appendix. Questionnaire).

### Conceptual framework

**Fig 2:**
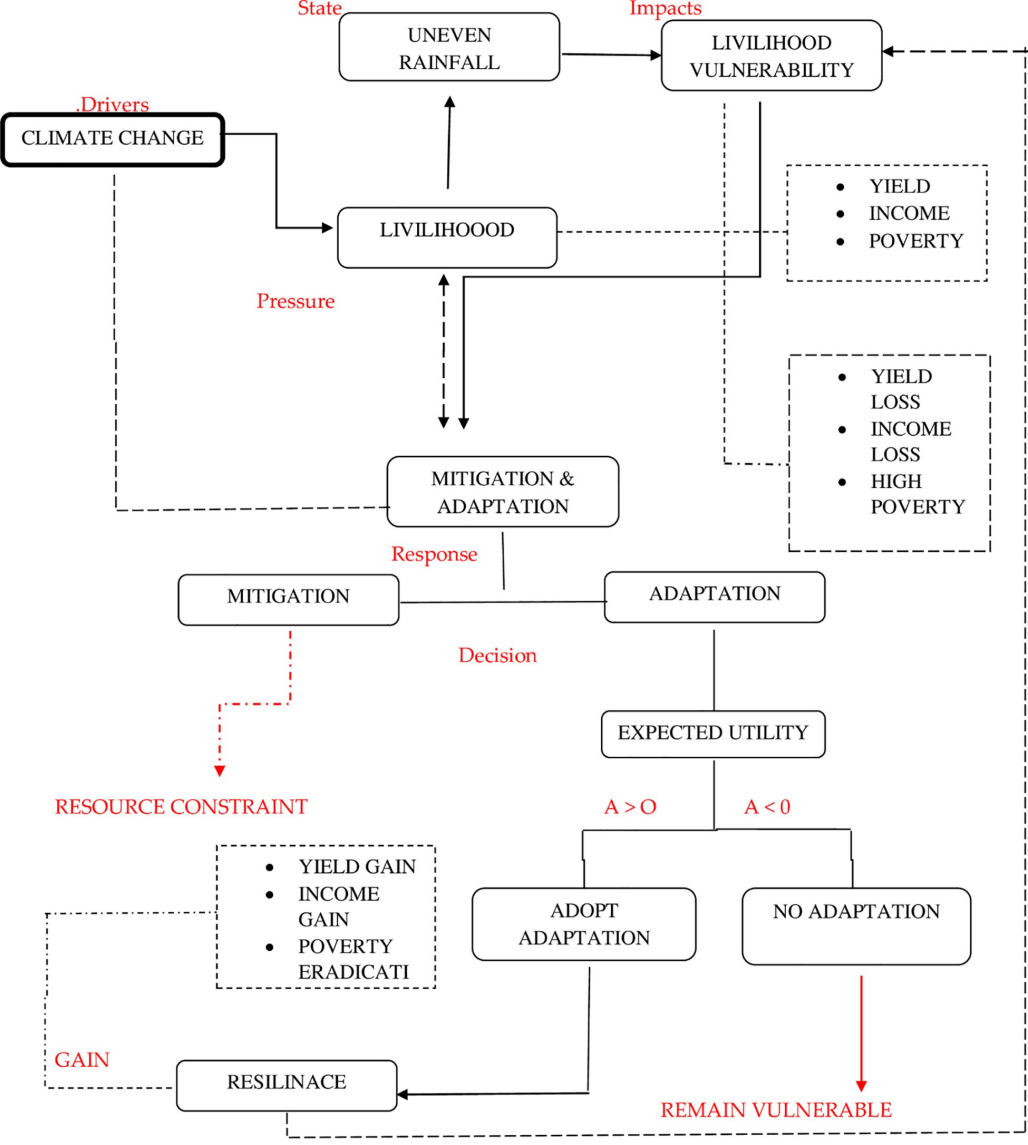
Conceptual framework of study (driver, pressure, state, impact, response, decision, and gain).

This study is primarily centered on the expected utility theory which states, a decision could be made based on the expected gain from participating adaptations in response to CC. Conceptual framework of this study given in Fig 2 that is based upon a further extension of Driving force, Pressure, State, Impact response (DPSIR) sustainability framework developed by [65]. Here, CC is a basic driver that exerts pressure on the livelihood of rural households; uneven rainfall is the state, which can have an impact on the livelihood of rural households in the form of a reduction in the farm yield, loss of income and poverty elevation. Response to this phenomenon is mitigation or adaptation to CC. Farmers take a decision, as they have the constraint to resources, and mitigation may not the appropriate strategy and adaptation becomes meaningful in this scenario. Adaptations are based on the expected utility, if utility from adoption is more than the utility from non-adoption, a farmer definitely would switch to an adaptation.

The actual amount of utility is hard to observe. So, a dummy for utility would be used with the value equal to 1, if a farmer adopts adaptation to CC, and its value would be 0 in the rest of the cases. Let us assume, the expected utility follows a latent variable $A_{1i}^{x}$ denotes the expected utility gained by adopting adaptation [49–51]. Whereas, $A_{0i}^{x}$ denotes the expected utility gain if he does not adopt. Therefore, the decision can be modeled in the index function as in the following Eq 1,

$$A_{i}^{x}=Z_{i}^{x}a+\mu i$$ (1)

$$A_{i}=1\;\mathrm{If}A_{i}^{x}>0\;\mathrm{and}\;A_{i}{=0\;\mathrm{If}\;A}_{i}^{x}\leq 0$$

Where $A_{i}^{x}$ is a dummy for expected utility, $Z_{i}^{x}a$ is a vector for the parameter of observed variable and *ϵ*_i_ is error term following the normal distribution. Here, the utility is expected to gain from adaptation in the form of an increase in the yield, income, reduction of poverty and resilience form vulnerability of climate. It is assumed that if a farmer adopts the adaptation then he may gain welfare, and resilience from vulnerability.

### Endogenous Switching Regression (ESR)

ESR is a parametric approach, which addresses the problem of selective biased and missing unobserved characteristics, which influence the decision to adopt the adaptation to CC [66–70]. It follows the two-stage analysis. In the first stage, the model estimates the selection equation to quantify the impact of determinates of adaptation. In the second stage, the outcome variable is estimated including inverse mill ratios to address the selective bias. By using the ESR model, we face two different regimes (Eqs 2 and 3),

$$Y_{i}^{1}=Z_{i}\beta_{i}+\varepsilon_{1}\;(\mathrm{Regime}\;1:\;A_{i}=1)$$ (2)

$$Y_{i}^{2}=Z_{i}\beta_{i}+\varepsilon_{2}\;(\mathrm{Regime}\;2:\;A_{i}=0)$$ (3)

Here *Y*_i_^1^ is the outcome of household if he adopts, and *Y*_i_^2^ is the outcome if he does not adopt, *Z*_i_ is vectors of observed explanatory factors including socioeconomics characteristics of CC indicators, and institutional facility that influences the outcomes. *β*_i_ is the parameter or coefficients to be estimated, *ε*_2_ is the disturbance term. Following [38, 66, 71, 72], we used full information maximum likelihood to estimate ESR and its selection equation for the outcome are given below.

$$\ln A=\sum_{i=1}^{N}=\left\{A_{i}\omega_{i}[\begin{array}{c} \ln\phi \left(\frac{Z_{i}^{x}a+\rho^{1}\mu_{i}\left(Y^{1}{}_{i}-Z_{i}\beta \right)/\sigma^{1}}{\sqrt{1-\rho^{21}\mu_{i}}}\right)+\ln\phi \left(\left(Y^{1}{}_{i}-Z_{i}\beta w\right)/\sigma_{1}\right)/\sigma^{1}+\\ \left(1-A_{i}\right)\omega_{i}\left[\ln\left(1-\varphi \left(\frac{(Z_{i}^{x}a+\rho_{2}\mu_{i}\left(Y^{2}{}_{i}-Z_{i}\beta \right)/\sigma^{2}}{\sqrt{1-\rho^{22}\mu_{i}}}\right)\right)\right]+\ln(\varphi \left(\left(Z_{i}^{x}a+\rho_{2}\mu_{i}\left(Y^{2}{}_{i}-Z_{i}\beta \right)/\sigma^{2}\right)/\sigma^{2}\right) \end{array}]\right\}$$ (4)

In Eq 4, *ω*_i_ is the optional weight of observation*ι*, *ρ*_1_*μ* = *σε*_1_*μ*_i_/*σε*_1_*σμ*_i_ is the correlation coefficient of error term of regime one, and selection equation, *ρ*_2_*μ* = *σε*_2_*μ*_i_/*σε*_2_*σμ*_i_ is the correlation coefficient of error term of regime two and selection equation in addition to it if *ρ*^2^_1_*μ* and *ρ*^2^_2_*μ* have alternative signs.

### Conditional expectations

Average treatment on treated (ATT), average treatment on untreated (ATU) and heterogeneity effect are estimated by conditional expectation [73] (Eqs 5 to 8):

$$E(Y_{1i}|A_{i}=1,Z_{i})=Z_{i}\beta_{i}+\sigma \varepsilon_{1}\mu_{i}\xi_{1i}$$ (5)

$$E(Y_{2i}|A_{i}=0,Z_{i})=Z_{i}\beta_{i}+\sigma \varepsilon_{2}\mu_{i}\xi_{2i}$$ (6)

$$E(Y_{1i}|A_{i}=0,Z_{i})=Z_{i}\beta_{i}+\sigma \varepsilon_{1}\mu_{i}\xi_{2i}$$ (7)

$$E(Y_{2i}|A_{i}=1,Z_{i})=Z_{i}\beta_{i}+\sigma \varepsilon_{2}\mu_{i}\xi_{1i}$$ (8)

Here, *E*(*Y*_1i_|*A*_i_ = 1,*Z*_i_) and *E*(*Y*_2i_|*A*_i_ = 0,*Z*_i_) represent the farmers who adopt the adaptation and do not adopt, respectively, and these indicate the actual expectation. *E*(*Y*_1i_|*A*_i_ = 0,*Z*_i_) and *E*(*Y*_2i_|*A*_i_ = 1,*Z*_i_) represent the farmers who do adopt the adaptation if they do not adopt the adaptation and if those who do not adopt the adaptation do adopt the adaptation, and these represent the counterfactual expected outcome.

Hence, ATT can be derived as:

$$ATT=E(Y^{1}{}_{i}|A_{i}=1,Z_{i})-E(Y_{2i}|A_{i}=1,Z_{i})$$ (9)

Moreover, ATU can be specified as,

$$ATU=E(Y^{2}{}_{i}|A_{i}=0,Z_{i})-E(Y_{1i}|A_{i}=0,Z_{i})$$ (10)

Yield has been taken in the form of maund per acre. Per capita income is calculated by dividing total household income per day by household size. Poverty status has been computed with the help of income (PKR) threshold given globally. The poverty gap is calculated by following Foster-Greer-Thorbecke, 1984 methodology [74]. The poverty gap is used to estimate the difference of poverty line with an actual income of a member of the society. All statistical analyses are performed in STATA software.

## Results

This section presents the results of the empirical estimation of determinants of CC adaptations, and trends of explanatory variables. Analysis of this article is divided into three parts. In the first, descriptive analysis is presented, the second part of the analysis is about the outcomes of ESR modeling and the last portion gives the conditional expectation investigations. Variables description of all explained and explanatory variables are given in Table 2. Whereas Table 3 depicts the summary statistics, and the mean difference between adapters and non-adapters are given in Table 4. Farmers’ perceptions about CC vulnerability are given in S 2. Datasets used for the study are also provided in supplementary information as S 3. Table 5 shows the results of likelihood ESR for the impact of CC adaptation. This table has four subpanels that describe the results of four different analyses by taking yield, per capita income, poverty status and poverty gap as dependent variables, while the same explanatory variables have been used for all four analyses, respectively. The results for the synergy analysis are given in Table 5.

**Table 2:** Description of selected variables.

Variable	Variable description	Variable type
AGE	Respondent’s age (years)	Independent variable
EDUCATION	The education level of the respondent (years)	Independent variable
FARMING EXPERIENCE	Farming experience (years)	Independent variable
FML SIZE	Family size (number of the household member)	Independent variable
FML SYSTEM	Family system (dummy variable with value 1 if nuclear, 0 otherwise)	Independent variable
FMLE PART	Female participation in farm operations (dummy variable with value 1 if yes, 0 otherwise)	Independent variable
CAR	Household owns the car(s) (dummy variable with value 1 if yes, 0 otherwise)	Independent variable
SMART PHN	Household use a smartphone for adaptations (dummy variable with value 1 if yes, 0 otherwise)	Independent variable
T LAND	Total agriculture land (acres)	Independent variable
IRRIGATION FACL	Irrigation facility availability (dummy variable with value 1 if yes, 0 otherwise)	Independent variable
TIME HHH	Time household head (HHH) spend on-farm (number of hours)	Independent variable
TIME FMH	Time other family members spend on the farm (number of hours)	Independent variable
LIVESTOCK	Household grazing livestock (dummy with value 1 if yes, 0 otherwise)	Independent variable
AGRI MCHNR	Household owns agricultural machinery (dummy with value 1 if yes, 0 otherwise)	Independent variable
NO LVSTK	Livestock units at household’s farm (number)	Independent variable
INCM LVSTK	Monthly income generated from livestock farming (PKR)	Independent variable
T EXP	Total monthly expenditure by the household (PKR)	Independent variable
HHMNTINCM	Monthly household income from all source (PKR)	Independent variable
AGRIANL INC	Household farm income in total (PKR)	Independent variable
AW CC	Awareness of CC (dummy 1 if yes 0 otherwise)	Independent variable
CNCR CC LOSS	Concerned about losses due to CC (dummy 1 if yes 0 otherwise)	Independent variable
FML EFC CC	CC indicators affected your farming practices (dummy 1 if yes, 0 otherwise)	Independent variable
AS CRDT	Access to credit for farming (Dummy 1 if yes 0 otherwise)	Instrumental variable
AS EXTN	Access to agricultural extension facilities (Dummy 1 if yes, 0 otherwise)	Instrumental variable
CC ADPR	The adapter of CC adaptation (dummy equal to 1 if yes and 0 otherwise)	Dependent variable
YIELD	Wheat yield in maund per acre, and 01 maund = 40 Kg	Dependent variable
PCINC	Per capita income of headcount (PKR)	Dependent variable
POVERTY	Poverty (dummy equal to 1 if yes and 0 otherwise)	Dependent variable
POVERTY GAP	Distance from income threshold	Dependent variable

**Table 3:** Summary statistics of variables of interest.

Variables	Mean	Std. Deviation	Minimum	Maximum
Age (year)	41.223	11.6232	25	80
Education (year)	7.11	4.562	0	16
Family Size (no.)	7.229	3.0843	3	22
Female participation	0.327	0.4697	0	1
Smartphone	0.607	0.4888	0	1
Total land (acre)	9.408	8.0485	2	25
Time spent on the farm by HHH (hours)	6.566	1.6522	3	10
Time spent on the farm by other family members (hours)	1.438	1.6672	0	6
Irrigation facility	0.699	0.459	0	1
Agricultural machinery facility	0.299	0.4581	0	1
Livestock units	0.873	0.3331	0	1
Awareness of CC	0.806	0.396	0	1
Access to credit	0.544	0.4986	0	1
Climate change adaptor	0.681	0.4666	0	1

Source: Authors’ calculation.

**Table 4:** Mean difference between adapters and non-adapters.

Variable	Variable description	Adaptors	Non-Adaptors	Difference
Age	Age (year)	42.543	39.957	2.586
Education	Education (year)	6.936	4.587	2.349
FML Size	Total family members of the household (no.)	7.096	7.413	-0.317
SMART PHN	Household use smartphone (dummy variable)	0.66	0.522	0.138
T LAND	Total agriculture land (acre)	9.426	3.293	6.133
TIME HHH	Time household head spend on the farm (hour)	6.947	7.326	-0.379
IRRIGATION FACL	Irrigation facility availability (dummy variable)	0.777	0.565	0.212
AGRI MCHNR	Farm machinery (dummy 1 if yes 0 otherwise)	0.362	0.196	0.166
LIVESTOCK	Household grazing livestock (dummy)	0.84	0.935	-0.095
AW CC	Awareness to CC (dummy)	0.915	0.587	0.328
AS CRDT	Access to credit (dummy)	0.585	0.478	0.107
AS EXTN	Access to extension (dummy with value 1 if yes and 0 otherwise)	0.628	0.609	0.019
YIELD	Wheat yield (maund per acre)	38.686	34.739	3.947

Source: Authors’ own calculation.

**Table 5:** Findings of full information likelihood ESR (impact of CC adaptation).

Dependent variable	Yield			Per Capita Income			Poverty Status			Poverty Gap		
Explanatory variable	Selection	Adapter	Non-adapter	Selection	Adapter	Non-adapter	Selection	Adapter	Non-adapter	Selection	Adapter	Non-adapter
	Coefficient	Coefficient	Coefficient	Coefficient	Coefficient	Coefficient	Coefficient	Coefficient	Coefficient	Coefficient	Coefficient	Coefficient
	(S.E)	(S.E)	(S.E)	(S.E)	(S.E)	(S.E)	(S.E)	(S.E)	(S.E)	(S.E)	(S.E)	(S.E)
**Age**	.0349 **	0.0164	-.104 ***	.0408 ***	-0.0109	0.0037	.0334***	0.0003	-.0044 **	.0294	-0.00222	.0023
	0.0083	0.0101	0.019	0.008	0.0152	0.0074	0.008	0.0021	0.0013	0.025	0.0028	0.026
**Fmlsize**	-.1365 **	.1058 **	-.453 **	-.135 ***	-.812 ***	-.1313 ***	-.138 ***	.0451 ***	-.0122 *	-.0.27 *	.0399 *	0.0480***
	0.0405	0.0388	0.113	0.036	0.0594	0.0403	0.032	0.007	0.0070	0.0973	0.0479	0.0020
**Timehhh**	-0.0116	.464 ***	-0.118	-0.010	.563 ***	.112 **	0.048	-.0188 *	-.0210 **	-.2415 **	0.015	-.0142
	0.0529	0.066	0.139	0.051	0.100	0.0524	0.055	0.0125	0.0086	0.111	0.015	0.015
**Seeds**	.0009 **	.002 ***	-0.0003	.001 ***	0.0004	-.0004 *	.0009 **	0.0001	0.0000	.002**	-0.0001	-0009
	0.0003	0.0004	0.0009	0.0003	0.000	0.0003	0.0003	0.0000	0.0000	0.000	0.0001	0.000
**Smartphn**	.3278 *	1.47 ***	-1.085 **	.3.668 **	1.216 ***	0.1314	.2928 *	-0.0515	-0.100	0.513	0.026**	0.0131
	0.1715	0.210	0.418	0.1678	0.314	0.158	0.164	0.0390	0.0258	0.775	0.022	0.042
**Irrigationfacl**	-.4563 **	-0.228	2.645 ***	-.339 *	-0.151	.089	-.297*	0.0067	0.0465	-0.362	0.0260	-.0.456
	0.1967	0.254	0.490	0.1989	0.384	0.183	0.192	0.0472	0.0337	0.480	0.051	0.048
**Education**	.0637 **	.096 **	.411 ***	.0658 ***	-0.083	.0521 ***	.0599 **	-.024 ***	-0.0047	0.0615	-0.0018	-.009
	.0218	0.033	0.052	0.019	0.0523	0.0207	0.019	0.0065	0.0034	0.0911	0.0052	0.010
**Tland**	-0.0241	.065 **	0.037	-0.022	.770 ***	.1662 *	-.030*	-.025 ***	-.0263 ***	-0.088	-.0644 ***	-.009 **
	0.0164	0.017	0.052	0.0172	0.0261	0.0195	0.0160	0.0032	0.0033	0.0832	0.0025	0.045
**Nolvstk**	0.0621	.0570 **	-0.0703	.0610 **	-0.0190	-0.035	.0681 **	0.0030	.0237 ***	-0.032	-.0025 ***	-.007**
	0.0263	0.0151	0.069	0.0243	0.0226	0.027	0.0208	0.0023	0.0044	0.013	0.0004	0.001
**Wheatarea**	.260 ***	-.084 *	-.316 **	.268 ***	-.5765 ***	0.0260	.300 ***	-.534 ***	-.0518***	.448**	-0.025	-.0051
	0.0424	0.048	0.139	0.0443	0.073	0.0543	0.042	0.071	0.009	0.168	0.023	0.0019
**Texp**	1.28	2.92	.0001 **	9.16	.00005 ***	2.83	-	-	-	0.000	1.95	-3.77**
	0.000	0.000	0.0000	0.00001	0.000	0.0000	-	-	-	0.000	3.36	1.41
**Ascredit (I.V)**	.620 **	-	-	.272 ***	-	-	.373 *	-	-	-	-	-
	0.245	-	-	0.1861	-	-	0.180	-	-	-	-	-
**Awcc(I.V)**	1.570 ***	-	-	1.581***	-	-	1.55***	.526***	0.4511		-	-
	0.215	-	-	0.239	-	-	.255	0.72	-0.039		-	-
**Asextn (I.V)**	.4739 *	-	-	-	-	-	-	-	-	-	-	-
	0.253	-	-	-	-	-	-	-	-	-	-	-
**Constant**	-5.201 ***	30.68	41.61	-5.91 ***	2.253	1.383	-5.44 ***	1.017 **	-1.386***	-8.12 **	0.700	.035
	0.978	1.232	2.350	0945	1.879	0.8549	0.9065	0.267	0.151	2.95	0.573	0.467
**Lns1**	-	0.538***	-	-	.954 ***	-	-	-1.133***	-	-	-2.68	-
	-	0.400	-	-	0.045	-	-	0.048	-	-	1.78	-
**R1**	-	1254	-	-	-.447 *	-	-	-0.454*	-	-	-1.08	-
	-	0.263	-	-	0.236	-	-	0.23	-	-	-	-
**Lns2**	-	-	0.884***	-	-	-0.233***	-	-	-1.985**	-	-	-2.84
	-	-	0.075	-	-	0.0566	-	-	0.0567	-	-	1.966
**R2**	-	-	-1.265***	-	-	-0.019	-	-	-0.047	-	-	-0.5416
	-	-	.2150	-	-	0.138	-	-	0.164	-	-	4.08
**Number of observation**	489			489			489			261		

The critical values are at the 10, 5 and 1 percent level, significance denoted by *, ** and *** respectively.

### Adoption of adaptations

Results showed that several explanatory variables would increase the likelihood of farmers’ decisions to adopt the adaptation to CC. Determining the overall effectiveness of adaptation solutions in agriculture is challenging because it is impossible to accurately enumerate and model all economically feasible options [60]. In general, adaptations have a significant association with age, education, farming experience, smartphone, total land, institutional facility, and farmers' knowledge about CC. Consequently, age and smartphone positively affect the decision to adopt with statistically significant values (see Table 5 for details). Similarly, education has a positive significant association with CC adaptation. In contrast to it, the coefficient of household family members depicted a negative association with the dependent variable. Moreover, the area of wheat cultivation shows a highly significant relationship with the dependent variable. Variable related to institutional facilities (i.e. access to credit and agricultural extension) also have a positive association with the CC adaptations.

### Impact of CC adaptation on yield

The coefficient of age is not statistically different from zero for the case of CC adaptors. In contrast to it, age showed a negative significant impact on the yield of non-adapters. Similarly, the coefficient of time HHH spend on the farm has a positive sign for the adapters but negative in case of non-adapters. Results showed that seed price would have a significantly positive correlation with the adopting adaptation. Moreover, using a smartphone for farm adaptations showed positive and significant impact with the wheat yield of adapters, in contrast to it, its negative association has been observed for the case of non-adapters. Results showed that education could have a positive significant impact with yield in both cases of respondents. Furthermore, the numbers of livestock and total land showed significant association with the dependent variable. Hence, it can be concluded from the results of Table 6 that adaptation shares much in common with resilience in preventing the harmful impacts of climatic variations, as ATT is 5.25 Kg if a farmer chooses to adopt the CC adaptation for the yield and ATU would be 3.29 if he does not adopt.

**Table 6:** Conditional expectation analysis.

Treatment effect	Decision rule		Average treatment effect (ATE)
	To adapt	Not to adapt	
**Yield**			
**ATT**	43.000	37.750	5.250***
**ATU**	39.15	38.080	1.068 ***
**Heterogeneity effect**	(B.H)^a^ 3.85	(B.H)^N^ -0.33	(T.H) 1.960
**Per capita income**			
**ATT**	6.673	2.720	3.9529***
**ATU**	1.420	3.930	-2.514***
**Heterogeneity effect**	(B.H)^a^ 3.953	(B.H)^a^ -1.210	(T.H) 6.4669
**Poverty status**			
**ATT**	0.3486	0.4077	-0.0590 ***
**ATU**	0.4679	0.9294	-0.4615 ***
**Heterogeneity effect**	(B.H)^a^ -0.119	(B.H)-0.521	(T.H) 0.402
**Poverty gap**			
**ATT**	0.0181	0.1536	-0.1355***
**ATU**	0.1244	0.2095	-0.0850***
**Heterogeneity effect**	(B.H)^a^ -0.106	(B.H)^a^ -0.055	(T.H)-0.0505

*, ** and *** denote 10%, 5% and 1% significance level.

(B.H)ª represents base heterogeneity of adapters.

(B.H)ᶰ depicts base heterogeneity of non-adapters.

T.H shows transitional heterogeneity.

### Impact of CC adaptation on per capita income

Results of per capita analysis depicted that family size is negatively related to per capita income of a household at 1% level of significance, for both, adapters and non-adapters. Another important variable, time spent by HHH on-farm has a significantly positive impact on the per capita income of households for both cases. The variable of smartphone use is also statistically different from zero and highly significant. Total land and education are positively associated with the dependent variable, although, there is no impact on education in the case of CC adapters. Results of synergy analysis (Table 6) showed that ATT is 9.95 and ATU is -2.514.

### Impact of CC adaptation on poverty

The finding of poverty analysis given in Table 5 implies that the age of HHH would negatively influence the poverty status of non-adapters. In addition to it, the family size would have a statistically significant and negative relationship with poverty. Another variable, time spent at the field, show a negative impact on poverty for both cases of adapters and non-adapters. Furthermore, both variables level of education and total land, depict a highly significant association with the dependent variable with negative signs. Impacts of synergistic effects of adaptation and resilience become visible when we make a comparison of ATT of adapters and non-adapter (Table 6), ATT is -0.324 in case a farmer who does not adopt the option of ‘chose to adapt’ and ATU is -0.286 in case if he chose the option ‘not to adopt the CC adaptation’.

### Impact of CC adaptation on the poverty gap

For the case of the poverty gap, findings showed that there is a negative association between the time given to farm by the HHH and the poverty gap. Total land and livestock are statistically significant at 1 and 5% level of significance for adapters and non-adapters, respectively. The coefficients of variables, education and availability of irrigation facility, are statistically different from zero in the case of non-adapters, with negative values. Moreover, farmers who choose to adopt the adaptations in case if they are non-adapter are better off with the ATU value of -0.085.

## Discussion

This section provided the rationale and discussion of selected variables’ outcomes. The results obtained appeared to be robust because most of the estimated parameter coefficients showed the expected signs. An in-depth analysis of the study explores that overall; our results are consistent with the previous literature. In the case of the coefficient of the education level of respondents, its value specifies its positive association with adaptation. Accordingly, an increase in the number of years of education of farmers would increase the willingness to accept the technological advancements in farming operations. Our results indicate that farmers who are more educated would be more productive and efficient, and eventually, they would generate more farm income and overcome their poverty as compared to the less educated ones, and this argument is in line with the findings of other relevant studies [e.g. 23, 37, 51, 54–56].

The negative signs of family size in case of CC adaptations, yield, and per capita income are probably due to an increase in the household members that would decrease the likelihood of adoption of adaptation and these results validated the outcomes of previous studies like [55, 75]. Whereas, positive signs of the coefficients of family size in case of poverty and poverty gap indicate that increasing family size would increase the poverty and ultimately it would make difficult for the farmers with more family members to reduce the poverty gap relatively, and similar argument has been presented by [76].

Furthermore, results suggest that usage of smartphone for farming purpose increases the possibility to adapt to cope with CC. We hypothesized that farmers utilize this smart technology to explore the ongoing scientific advancements, information of changing climate and self-projection of future climate risks. In addition to the above, smartphones provide updated to the farmers about several adaptation strategies being applied to the agriculture sector around the globe. Coefficient of smartphone use is statistically different from zero and has a positive sign in case of adapter but negative for non-adapters, showing that adapter uses smartphone to get an update on climate risk and farm-level adaptation measures. In contrast, it is assumed that non-adapters are conventional and proportionately less efficient, and does not much rely on such technologies. Further, the smartphone showed a positive significant impact on per capita income headcount in the case of adapters. Per capita income headcount has strong linkage with the crop productivity. Meticulously, it can be said that an increase in the use of smartphones can help in increasing the mean net farm returns, income, and poverty reduction, and results of [77] are consistent with our findings.

Moreover, the findings of the study showed that an increase in age would increase the chances to adopt the CC adaptations. Inversely, the negative sign of the coefficient for wheat's yield specifies that with the increasing age, the capacity to work also goes down and these arguments are maintained by [51, 55, 68, 78], conversely, outcomes of [79] opposed this. Results show that total landholding and time spent by the HHH on-farm could have a positive and significant impact on the yield of adapters, implying an increase in the values of these both parameters would increase farm productivity overtime. Similar results have been observed for the case of total land for per capita income of headcount for both categories of farmers i.e. adapters and non-adapters, and through this finding our study validate the outcomes of a similar study by [37].

Results further showed that conditional expectation and ATT are statistically significant. The value of ATT indicates, those farmers are well off who have been treated by CC adaptation than those who are untreated. In conclusion, the study shows that overtime climatic variations are significantly exacerbating poverty and inequality. Thus, policymakers are well recommended to include the adaptations as potentially contributing factors, when designing policies to alleviate poverty and inequality. In general, findings of the study depicts that there is deep interaction between CC adaptation and resilience from livelihood vulnerability, as adapters are getting better farm outcomes and they are more well off than the non-adapters. The key determinant of individuals, households, or communities’ adaptive capacity is to reduce risk, cope with, and adapt to increased risk level with farm resource portfolio. There are close linkages between vulnerability and resilience, and resilience is basically about the expanding and sustaining their farm resource [64, 80]. It is noted that if farmers who are adopters of adaptation, had not adapted, would be more vulnerable to climate variabilities than their current economic state. Hence, adopting adaptation is a meaningful tool for resilience from livelihood vulnerability.

## Conclusions

The present study analyzes the synergy between CC adaptation and resilience from livelihood vulnerability. The study used data from the four districts of the south part of the Punjab province of Pakistan. An intensive field survey of farming households is carried out. ESR model is employed for the analysis purpose. Results of the study reveal that education, usage of smartphone, and total land holding of the farmer are the main factors influencing the likelihood of adaptation to CC and above-said factors are also contributing positively towards yield, per capita, income and poverty indices found.

It can be concluded from the results of this article that education is the key factor in developing human capital. An increase in the level of education is indirectly helping the growers in enhancing farm net return through improved yield and income, and alleviation of poverty. Education builds the capacity to make a rational decision at the right time, hence they adopt the adaptation [81, 82]. Consequently, educated farmers are more productive and better off in monetary terms than their counterparts. This study also concluded that family size is one of the dilemmas, and increasing family size worsens the welfare gains. Furthermore, the study concludes that landholding and using a smartphone for updated agriculture-related information have a notable role in building adaptive capacity and a farmers’ resilient livelihood. It can be said from the study findings that smartphones, adaptive capacity, and farmer resilience are deeply interlinked with each other. Therefore, a farmer using a smartphone would have more knowledge about the new adaptation techniques and use them to moderate farm loses [61].

The most imperative conclusion of this study is the ATE established in the study. Results of ATE on treated showed that farmers who adopt are better off than the other due to potential gains from adaptation measures, and 14% of farm production of adapters is higher than the non-adapters that could be due to adaptations. The per capita income of adapters is also higher than the other groups. Adaptation contributes to the resilience of CC vulnerability [83, 84] and our findings validated the results of other regional-level studies [3, 83–85]. The ATE reflects the effectiveness of adaptation strategies to abate CC vulnerabilities. An increase in the output of crops will help in increasing the living standard of the rural household through income increase and poverty reduction. The study shows that adaptations play a supportive role in recovering the vulnerability that substantiates the synergy between adaptation and resilience to CC vulnerability.

**Fig 3:**
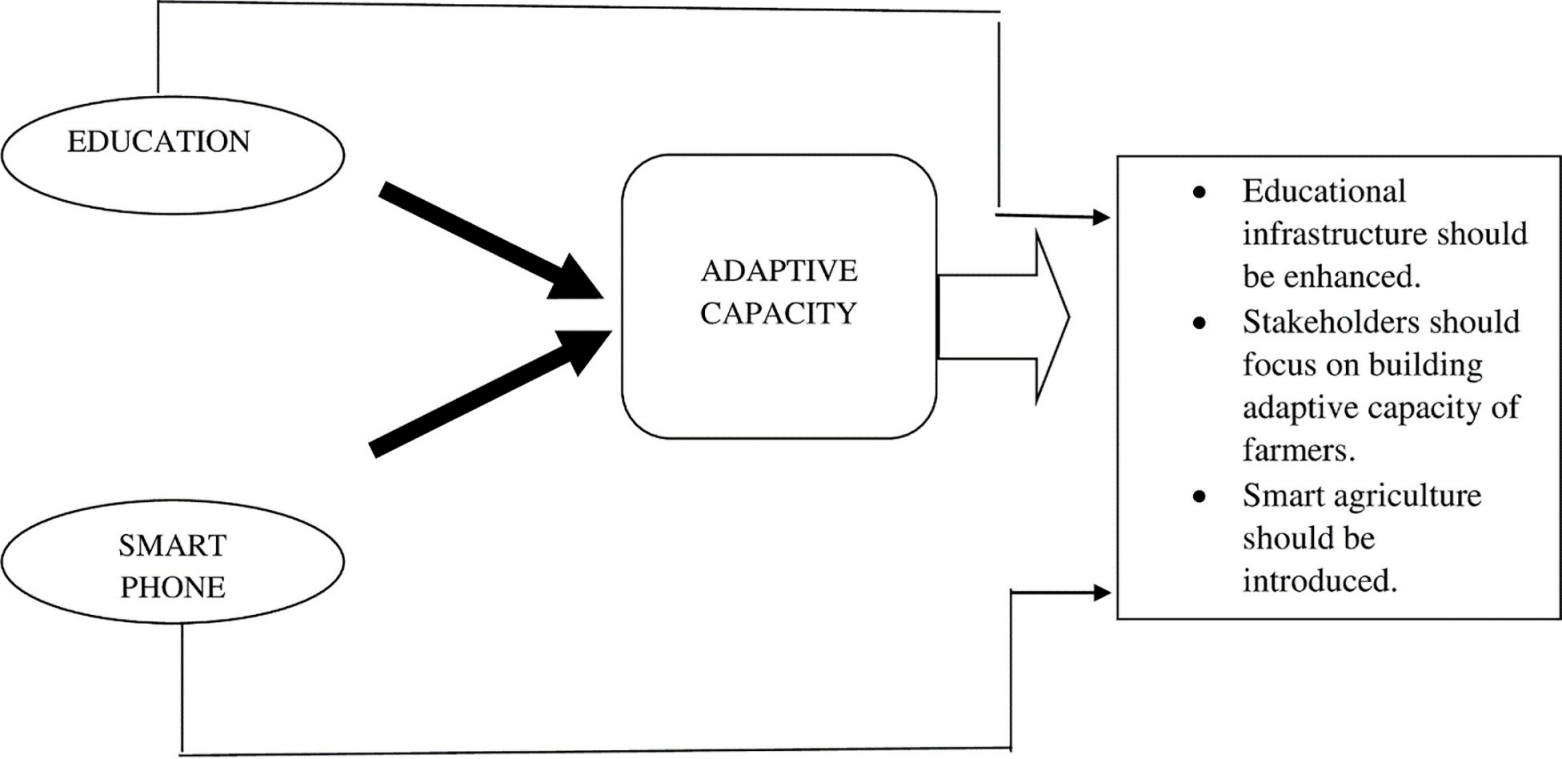
Policy implication framework.

This study has successfully archived its stated objectives. Study yields a wide spectrum of policy options for policymaking, and practice-oriented solutions to cope with and adapt climate change (Fig 3). Firstly, the study suggested the need for proper education infrastructure in rural areas for farming communities to understand modern problems with modern solutions. Secondly, concerned stakeholders are suggested to provide the facility of smart technology in the rural areas to equip the farmers with timely updates about CC and new adaptation techniques. Policymakers are also suggested to provide their urgent concentration on the adaptive capacity building of the farmers.

## Supporting information

S1 Appendix Questionnaire. (DOCX)

S2 Appendix CC vulnerability from the respondents’ perspective. (DOCX)

S3 Appendix Datasets. (XLSX)
