# Supplementary material for: Synergy between adaptations and resilience of livelihood from climate change vulnerability: A group-wise comparison of adapters and non-adapters
Source: PLoS One. 2020 Aug 13;15(8):e0236794. doi: 10.1371/journal.pone.0236794 (PMC7425974; doi:10.1371/journal.pone.0236794)
Supplement: S1 Appendix — (DOCX) [file pone.0236794.s001.docx]

**Synergy between adaptations and resilience of livelihood from climate change vulnerability**

**Serial no…………………….**

**Section 1**

**Demographic status**

Q Are you household head 1. Yes 2. No

Section 1: household demographics

Q. Name of Farmer: ………………………………….. Q. Gender: 1. Male 2.Female

Q. Age: …………………years. Q. Marital status: 1. Married 2. Unmarried

Q. Occupation: 1. Farming 2. Other than farming Q. Farming Experience …………Years

Q. Secondary Occupation: ………………………… Q. Education: …………………years

Q. Technical education ………………… Q. Village Name: ………………………………… Q. Tehsil: ………………………………. Q11. Contact Number: …………………………….

**Section 2: Socioeconomics characteristics**

1. **Family information**

Q. Family Size: …………………… Q. family members ages : 1 to 18 ………….

Q. Family system 1.nuclear 2.joint 18 to 30 ………….

Q. Female participation yes No 30 to 60 ………….

Q. School going children: …………………… 0bove 60 ………….

Q. College going children: …………………….

Q. Do you own a car 1.Yes 2. No Q. Do you use smart phone 1.Yes 2. No

Q. Resident: 1. own 2. Rented

Q. House type: Cemented house mudded house

Q. Do you have Health facility 1.Yes 2. No

Q. Do you have sanitation facility 1.Yes 2. No

**2. Land Information**

Q. Total Land: ………….……….acre Q. Land Owned ………………………acre

Q. Land on lease ……………… acre Q. Land use for agriculture ………………...acre

Q. land use for other purpose ………………………… acre.

Q. Crops grow: Rice Wheat Sugarcane cotton maize

Other (specify) …………………….

Total area for wheat crop ………………acre total area for rice crop ………….. acre

Total area for sugarcane…………………acre oher crops area……………….acare

Q. No of hours spend in field by household head …………………hours

Q. No of hours spend in field by other member of household …………………. Hours

Q. Do you have irrigation facility 1. Yes 2. No

Q. (If yes) What is your source of irrigation?

1. Canal 2. Shallow tube well 3. Deep tube well 4. Drip irrigation

5. Sprinkler irrigation

Q. (if no) is your land is under rain fed area? 1. Yes 2. No

Q. Is your village having infrastructure (multiple answer is possible)

1.Roads 2. Cold storage 3. Market of grain 4. Other ………………..

Q. Do you own any agriculture machinery? 1. Yes 2. No

Q. If (yes) which of the following you own?

1. Tractors 2. Harvesters 3. Harrow 4. Planter 5. thresher 6.manure spreader 7. Other specify ………………….

Q. If (No) how you operate your farm?

1. Use of labor 2. Use of animals 3. Rent machineries

Q. Do you keep livestock 1. Yes 2. No

Q. If (yes) number of livestock …………………..

| Cow | Buffalo | Goat | Hen | Male & yungling |
| --- | --- | --- | --- | --- |
|  |  |  |  |  |

Q. what is your purpose of keeping livestock

1. Self-consumption 2. Sale of milk 3. For transportation 4. For sale meat

5. For sale livestock

Total earings from livestock ………………..Rs (monthly)

**3. Income & expenditure**

**Income**

Q. Major source of income: 1. Agriculture crops 2. Other (specify)…………………..

Q. Annual income: ……………………. Rs Q. Monthly income: ……………………Rs

Q. Number of household employed………… Q. Number of household unemployed…………...

Income from other family members

| Sr no | Male income | Female income | Children income | Other |
| --- | --- | --- | --- | --- |
| 1 |  |  |  |  |
| 2 |  |  |  |  |
| 3 |  |  |  |  |
| 4 |  |  |  |  |

**Expenditure household ( monthly)**

| Food | Heath | Housing | Clothing | Saving | Other |
| --- | --- | --- | --- | --- | --- |
|  |  |  |  |  |  |

Total expenditure ……………………..

**Section 4 Climate change information and perception**

Q. Do you know what climate change is?

1. Yes 2. No

Q. If yes how do you know about climate change?

1. Self-assumption 2.word of mouth 3. T.v or radio 5. Smartphone 5. Agriculture facilitation center 6.Government pamphlet or reports

If NO do you have an idea of following changes in weather?

1. Temperature rise 2. Erratic rainfall 3. Increasing number of heat waves 4. Precipitation 4.droughts

Q. What climate change indicator you think effected your farming practices during last 10 years?

Temperature rainfall storms droughts precipitations

Q. Are concerned/worried about losses due to climate change?

1. Yes 2. No

Q. Do you think you negatively contribute in climate change?

1. Yes 2. No

Q. If yes then how?

1. Using more fertilizers 3. Using carbon emission products (vehicles) 2. Cutting trees

Q. Do you think you contribute to reduce climate change?

1. Yes 2.No

Q. If yes how? 1. Planting trees 2. Using efficient products (vehicles) 3. Using organic fertilizer

Q. Do think your family member affected by climate change

1. Yes 2.No

Q. If yes how?

1. Reduction in income 2. Increase in poverty 3. Cereal scarcity 4. Loss in productivity 5. Food insecurity 6. Other specify ………………….

Q. Do you think climate change effect can be controlled?

1. Yes 2.No

**Section 5 Climate change loss from farmer precipitations**

**(1 very high 2 high 3 modrate 4 less 5 very less)**

| Sr no | Question | 1 | 2 | | 3 | | | 4 | | 5 |
| --- | --- | --- | --- | --- | --- | --- | --- | --- | --- | --- |
| 1 | losses due to change in temperature according to your perception during last 10 years? |  | |  | |  |  | |  | |
| 2 | How much you lost due to change in rainfall according to your perception during last 10 years? |  | |  | |  |  | |  | |
| 3 | How much you lost due to change in heat storms according to your perception during last 10 years? |  | |  | |  |  | |  | |
| 4 | How much you lost due to change in precipitation according to your perception during last 10 years? |  | |  | |  |  | |  | |

**Section 3 Livelihood vulnerability due to climate change assessment**

(1. Very high 2. High 3. Modrate 4. Less 5. Very less)

| **Sr. no** | **Question no** | **1** | **2** | **3** | **4** | **5** |
| --- | --- | --- | --- | --- | --- | --- |
| 1 | Is there any loss to income? |  |  |  |  |  |
| 2 | Is there any loss to food security of your household? |  |  |  |  |  |
| 3 | Is there any loss to crop productivity? |  |  |  |  |  |
| 4 | Is there any loss in calories intake of your family? |  |  |  |  |  |
| 5 | Is there any loss in protein intake of your family? |  |  |  |  |  |
| 6 | Is there any loss in health facility of your family? |  |  |  |  |  |
| 7 | Is there any loss to education facility of your family? |  |  |  |  |  |
| 8 | Is there any loss to livelihood pattern of your household? |  |  |  |  |  |
| 9 | Is there any change in your food consumption pattern? |  |  |  |  |  |
| 10 | Is there any change in your expenditure pattern? |  |  |  |  |  |
| 11 | is there any household member face heat stroke due to extreme temperature? |  |  |  |  |  |
| 12 | Is there any household member got sick due to extreme rain pattern? |  |  |  |  |  |

**Section 7 (Institution facility of farmers)**

| Serial no | Questions | Yes | No |
| --- | --- | --- | --- |
| 1 | Do you have access to credit? |  |  |
| 2 | Do you have access to extension? |  |  |
| 3 | Do you have access to crop insurance? |  |  |
| 4 | Do you have access to technical education? |  |  |
| 5 | Do you use smart phone for knowing agriculture technique? |  |  |
| 6 | Do you learn new agriculture technology? |  |  |
| 7 | Do you have any government support to offset climatic damages |  |  |
| 8 | Do you have any subsidy from government |  |  |

**Section: 8 (Climate change adaptation)**

Q. Are you aware of climate change adaptation?

1. Yes 2. No

Q. If yes how do you know about climate change adaptation?

1. Self-assumption 2.word of mouth 3. T.v or radio 5. Smartphone 5. Agriculture facilitation center 6.Government pamphlet or reports

Q. Which type of adaptation technique you use to adopt?

1. on-farm adaptation 2. Off-farm adaptation

| **Information on adaptation techniques used by farmers** | | | | | | | | | |
| --- | --- | --- | --- | --- | --- | --- | --- | --- | --- |
| On farm adaptations | | | | | | | | | |
| Sr no | Questions | Planting shaded tree | Changing crop verity | Changing sowing data | Use hybrid seeds | Use of temperature tolerant seeds | Use of water tolerant seeds | Use of drough tolerant seeds | Artificial water course |
| 1 | which adaptation technique you used for change in temperature |  |  |  |  |  |  |  |  |
| 2 | Q. which adaptation technique you used for change in rainfall? |  |  |  |  |  |  |  |  |
| 3 | which adaptation technique you used for change in drought? |  |  |  |  |  |  |  |  |
| 4 | which adaptation technique you used for change in heat storm? |  |  |  |  |  |  |  |  |
| 5 | Which adaptation technique you used for change in precipitation? |  |  |  |  |  |  |  |  |
| Off farm adaptation | | | | | | | | | |
| Sr no | Questions | Migration | | Lease land area to other farmer | | Changing land use | | Finding new source of income | |
| 6 | Which of following off farm adaptation you use? |  | |  | |  | |  | |

**Section 6 (Farmer reliance after adaptation)**

| Sr no | Questions | Yes | No |
| --- | --- | --- | --- |
| 1 | Does using hybrid increase yield of your crops? |  |  |
| 2 | Does changing sowing date save your crops from suffer from climate extremes? |  |  |
| 3 | Does digging water course save crops by extracting rainwater out from field? |  |  |
| 4 | Does adopting adaptation increase your income |  |  |
| 5 | Does adopting adaptation increase your yield |  |  |
| 6 | Does adoptiang adaptation increase your food security |  |  |
| 7 | Did you gain welfare after adopting adaptation techniques |  |  |

**Section 9 (Land revenue and expenditure)**

| Operational cost Per crop | Land prepration Rs | | | | Seeds Rs | Sowing wage Rs | | Fertilizer Rs | Irrigation Rs | | Harvesting wage Rs | | Transportation wage Rs |
| --- | --- | --- | --- | --- | --- | --- | --- | --- | --- | --- | --- | --- | --- |
| Crops |  | | | | | | | | | | | | |
| Rice (per acre) |  | |  | | |  |  | |  | |  | |  |
| Sugercane (per acre) |  | |  | | |  |  | |  | |  | |  |
| Wheat (per acre) |  | |  | | |  |  | |  | |  | |  |
| Other crops (per acre) |  | |  | | |  |  | |  | |  | |  |
| **Revenue** | | | | | | | | | | | | | |
| Revenue per crop | | Yield (kg) | | grain use for seeds (kg) | | | | | Price per 40 kg | Total revenue Rs | | Net income Rs | |
| Crops | |  | | | | | | | | | | | |
| Rice (per acre) | |  | |  | | | | |  |  | |  | |
| Sugercane (per acre) | |  | |  | | | | |  |  | |  | |
| Wheat (per acre) | |  | |  | | | | |  |  | |  | |
| Other crops (per acre) | |  | |  | | | | |  |  | |  | |
