# Supplementary figures and images for: Synergy between adaptations and resilience of livelihood from climate change vulnerability: A group-wise comparison of adapters and non-adapters
Source: PLoS One. 2020 Aug 13;15(8):e0236794. doi: 10.1371/journal.pone.0236794 (PMC7425974; doi:10.1371/journal.pone.0236794)

**Appendix 1: Climate change vulnerability from the respondents’ perspective**


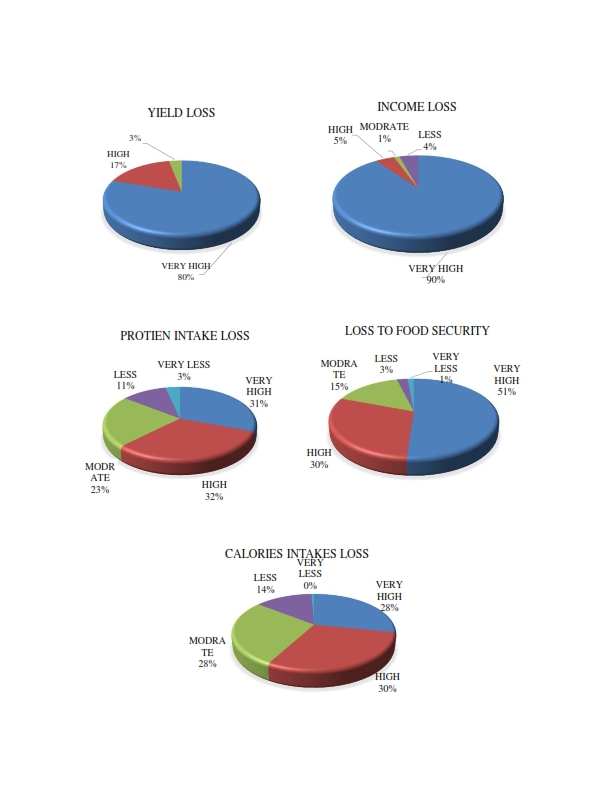

Supplement: S2 Appendix — (DOCX) [file pone.0236794.s002.docx]
